# A reduction in serum IFN-γ, IL-1β, IL-6, and GM-CSF serves as potential markers for a positive outcome to lumbar epidural steroid injections

**DOI:** 10.1371/journal.pone.0329552

**Published:** 2025-08-22

**Authors:** Pornpan Chalermkitpanit, Weerasak Singhatanadgige, Wicharn Yingsakmongkol, Teerachat Tanasansomboon, Manassanan Jitjumnong, Sittisak Honsawek

**Affiliations:** 1 Pain Management Research Unit, Department of Anesthesiology, Faculty of Medicine, Chulalongkorn University, King Chulalongkorn Memorial Hospital, Bangkok, Thailand; 2 Department of Orthopedics, Faculty of Medicine, Chulalongkorn University, King Chulalongkorn Memorial Hospital, Bangkok, Thailand; 3 Center of Excellence in Biomechanics and Innovative Spine Surgery, Faculty of Medicine, Chulalongkorn University, Bangkok, Thailand; 4 Department of Orthopedics, Samut Sakhon Hospital, Samut Sakhon, Thailand; 5 Center of Excellence in Osteoarthritis and Musculoskeleton, Department of Biochemistry, Faculty of Medicine, Chulalongkorn University, King Chulalongkorn Memorial Hospital, Bangkok, Thailand; National Institute of Child Health and Human Development (NICHD), NIH, UNITED STATES OF AMERICA

## Abstract

**Introduction:**

This study evaluated serum inflammatory cytokine levels before and after transforaminal epidural steroid injections (TFESI) to assess their potential as biomarkers for predicting favorable outcomes in patients with lumbosacral radiculopathy.

**Methods:**

All 120 eligible patients diagnosed with lumbosacral radiculopathy underwent blood collection 1) before TFESI and 2) two weeks after the procedure. A 5.0 mg dexamethasone mixture with local anesthetics was injected at each epidural level. This study defined a favorable response as a pain reduction of more than 50% at the 2-week follow-up. Serum inflammatory cytokines between good and poor responders were analyzed. Data collection included pain characteristics, clinical presentations, and radiographic findings.

**Results:**

Positive responses were observed in 73 out of 120 patients (60.8%). Patients with favorable responses demonstrated a significant reduction in serum IFN-γ, IL-1β, IL-6, and GM-CSF concentration at 2 weeks after TFESI compared to their baseline levels. However, mean differences in serum inflammatory cytokines post-TFESI showed no significant variation between two groups. The duration of pain symptoms was associated with increased levels of IFN-γ and decreased levels of MMP7. The increased serum IL-6, GDF-15, and GM-CSF exhibited a significant correlation with clinical presentations. The higher level of serum IFN-γ significantly correlated with the severity of HNP (r = 0.436, P = 0.039) while the elevated serum concentration of MMP7 exhibited a significant correlation with the presence of foraminal stenosis (r = 0.436, P = 0.045).

**Conclusion:**

The decrease in serum IFN-γ, IL-1β, IL-6, and GM-CSF levels post-TFESI was a promising biomarker for a favorable response in patients with lumbosacral radiculopathy.

## Introduction

A lumbar epidural steroid injection (ESI), administered near the targeted spinal nerve root, reduces inflammation and alleviates pain. The effectiveness of ESI varies within a range of 29% to 84%, depending on the spinal pathology [1–5]. A recent meta-analysis revealed that ESI presented the most compelling evidence (Level I) for treating pain from disc herniation, whereas the evidence level was IV for radicular pain caused by central stenosis or post-laminectomy pain [6]. One of the primary concerns of ESI is the dose-dependent suppression of the immune system, which poses the greatest risk for individuals with concurrent medical conditions or immunosuppression. Due to the COVID-19 pandemic, there has been increased apprehension about the risks associated with steroid injections. Hence, it is crucial to identify specific parameters for predicting the efficacy of ESI to ensure the appropriate selection of patients for this treatment.

Current research emphasizes the activation of macrophages and monocytes within the herniated disc, triggering the release of inflammatory cytokines, which in turn initiate the process of matrix remodeling and neovascularization [7]. This phenomenon highlights the significance of the inflammatory process in the context of lumbar disc herniation. In 2011, Golish et al. found an association between the presence of the fibronectin-aggrecan complex in the epidural lavage fluid and the achievement of significant pain relief following ESI [8]. This complex is a product of the degradation of structural proteins and fragments found in degenerative disc disease. Furthermore, among the inflammatory cytokines detected in epidural lavage fluid, a strong correlation was observed between interferon-gamma (IFN-γ) and the ability to predict 3-month pain reduction following ESI [9]. Interferon-gamma, an inflammatory cytokine, serves as the primary activator of macrophages. In clinical settings, serum high-sensitivity C-reactive protein (hs-CRP) is commonly utilized as a marker of inflammation and tissue damage. The study by Ackerman 3^rd^ et al. revealed that a higher level of hs-CRP before receiving ESI was associated with a lesser improvement in pain scores at 6 weeks [10]. However, data on the use of serum inflammatory cytokines as biomarkers for predicting the effectiveness of ESI remain limited.

This study emphasized additional critical inflammatory cytokines linked to macrophage activation, such as interleukin-1β (IL-1β), interleukin-6 (IL-6), tumor necrosis factor-α (TNF-α), matrix metalloproteinase-7 (MMP-7), and granulocyte-macrophage colony-stimulating factor (GM-CSF). It also investigated neovascularization-related cytokines, including vascular endothelial growth factor (VEGF) and growth differentiation factor 15 (GDF-15). This prospective analytical study investigated changes in serum inflammatory cytokines before and after TFESI to determine their potential as predictors of favorable treatment outcomes and the correlation between serum cytokines and the severity of magnetic resonance imaging (MRI) findings in patients experiencing lumbosacral radiculopathy.

## Materials and methods

Patients aged 20 years and older, with a documented history of low back pain and radicular pain, diagnosed with lumbosacral radiculopathy, were recruited between August 2020 and December 2022 upon approval of the Institutional Review Board of King Chulalongkorn Memorial Hospital, Bangkok, Thailand (IRB number 321/63). Written informed consent was obtained from all patients included. The inclusion criteria included patients who did not respond to conservative treatments, including medications, physical therapy, rehabilitation, and activity modification, as evidenced by a pain score upon movement of more than 4 out of 10 on a numeric rating scale (NRS 0–10). Radicular pain was defined as neuropathic, exhibiting a specific dermatomal pattern linked to the involvement of one or two lumbar nerve roots. This correlation was established through MRI findings, which indicated either disc herniation or degenerative spinal stenosis, including central, lateral recess, and foraminal stenosis as the primary diagnosis. The exclusion criteria encompassed individuals who had undergone previous lumbar spine surgery, experienced compression fractures or trauma, or displayed red flag symptoms such as progressive weakness, infection, tumor, or bowel/bladder issues. Additionally, patients with diagnoses of systemic inflammatory diseases (such as rheumatoid arthritis, inflammatory arthritis, ankylosing spondylitis, etc.), those with contagious diseases (including COVID-19 infection), individuals allergic to medication, suffering from depression, taking oral steroid medications, the current use of nonsteroidal anti-inflammatory drugs (NSAIDs), or having a history of prior epidural steroid injections within the last three months were also excluded from participation.

All eligible patients underwent venous blood collection at two-time points: initially, before the ESI, and subsequently, two weeks after the intervention. The analysis of inflammatory cytokines included assessments of hs-CRP, IFN-γ, IL-1ß, IL-6, TNF-α, MMP-7, GM-CSF, VEGF, and GDF-15. Subsequently, TFESI was conducted under fluoroscopic guidance by a pain specialist in an outpatient setting. All patients were positioned in a prone posture under standard monitoring. The procedure was performed utilizing local anesthesia, employing a 22-gauge Quincke spinal needle for subpedicular approaches while maintaining a sterile technique. The needle was carefully advanced, achieving its final placement as confirmed in the lateral view. To ensure proper needle placement and the absence of vascular or intrathecal patterns, a contrast medium (Omnipaque^®^) was injected in the anteroposterior view, confirming its spread into the epidural space. The mixture containing 5.0 mg of preservative-free dexamethasone in 1.5 ml of 2.0% lidocaine and 0.5 ml of 0.5% bupivacaine was administered at the indexed epidural level. A maximum dose of 10 mg of dexamethasone was targeted at the index level. Following the procedure, each patient experienced a pain reduction exceeding 90% while in the post-anesthetic care unit (PACU), indicating that the injection effectively addressed the specific spinal pain pathology.

All patients were instructed to assess their pain levels using an NRS ranging from 0 to 10 and to complete the Oswestry Disability Index (ODI) questionnaire at several time points: initially, before the TFESI, and subsequently, at 2 weeks, 1 month, 2 months, and 3 months following the procedure. For the 2-week follow-up evaluation, a favorable response to the TFESI was defined as a reduction in pain upon movement exceeding 50.0%, signifying a clinically meaningful improvement. This percentage was calculated by subtracting the pre-TFESI score from the post-ESI score and then dividing it by the pre-TFESI score. Patients who did not meet these criteria were classified as poor responders. A single TFESI injection was administered, and its impact was monitored until it resulted in less than 50% pain reduction. All patients were interviewed monthly by phone and visited our clinic every 3 months until 12 months after the procedure, regardless of whether they required surgical intervention. Data collection encompassed various parameters, including patient baseline characteristics, the duration of pain, body mass index (BMI), the occurrence of neurogenic claudication, the positive result of the straight leg raising test (SLRT), and the presence of motor power deficits. The assessment of MRI imaging by two certified radiologists involved several aspects, such as evaluating the extent and location of disc herniation according to the study reported by Fardon et al. [11], identifying the degree of central canal stenosis (measured by cross-sectional area and classified as mild, moderate, or severe) [12], grading the degree of foraminal stenosis [13] and nerve root compromise [14], and noting the presence of spondylolisthesis. The primary outcome of interest was comparing serum inflammatory cytokine levels between individuals classified as favorable responders and those identified as poor responders. Additionally, the secondary outcomes included the comparison of clinical manifestation and MRI findings between individuals categorized as favorable responders and those classified as poor responders, as well as evaluating the correlation between the level of cytokines, the intensity of pain, and the extent of spinal pathology observed in MRI findings.

### Laboratory methods

Ten-millimeter venous blood samples were collected into ethylene diamine tetra-acetic acid (EDTA) tubes. The serum was separated by centrifugation, followed by freezing at −20°C to facilitate subsequent assay analysis. To quantify hs-CRP, IFN- γ, IL-1β, IL-6, TNF-α, MMP-7, GM-CSF, VEGF, and GDF-15 concentrations in serum, double-blind assessments were performed using commercially available sandwich enzyme-linked immunosorbent assay (ELISA) kits, following the manufacturer’s recommended protocol. These ELISA kits were sourced from R&D Systems Bio-Techne Corporation, based in Minneapolis, USA. The minimum detectable dose (MDD) of human hs-CRP, IFN-γ, IL-1β, IL-6, TNF-α, MMP-7, GM-CSF, VEGF, and GDF-15 was less than 0.08 ng/mL, 2.56 pg/mL, 1.00 pg/mL, 0.70 pg/mL, 6.23 pg/mL, 0.094 ng/mL, 3.00 pg/mL, 9.00 pg/mL, and 0.95 pg/mL, respectively.

### Statistical analyses

The sample size was determined based on the “10 events per covariate” rule. Since the primary outcome of this study involved nine cytokine predictor variables, a minimum of 90 events was required. Based on a previous study [5], which reported an 84.0% proportion of favorable responses to ESI, the study aimed to enroll 107 participants. To accommodate potential dropouts, an additional 10% was included, resulting in a total sample size of 120 participants.

An unpaired *t*-test was employed to compare continuous data, while a Chi-square test was utilized to compare categorical data between the two responder groups. Additionally, a paired *t*-test was conducted to assess the differences between the pre-serum and post-serum levels of individual cytokines. Descriptive data were presented as the mean along with its standard deviation (SD) for continuous variables and percentages for categorical data. Univariate logistic regression analysis was initially conducted to assess the primary outcome. Odds ratios (ORs) and 95% confidence intervals (CIs) were calculated to evaluate the reliability of the estimates. Subsequently, multiple logistic regression analysis was employed to identify individual predictors, using variables with a p-value below 0.1 from the univariate analysis. A repeated measure analysis of variance test was conducted to examine the pain score and ODI over time between the two responder groups. Correlations among serum cytokines, pain scores, ODI, pain clinical presentations, and MRI findings were analyzed using Pearson correlation or Spearman rank correlation. The box plot depicting the correlation between pain clinical presentations and serum inflammatory cytokines displayed the median and interquartile range (IQR) spanning from Q1 to Q3. The chi-square test was used to compare the probability of receiving surgery between the 2 groups. Data analysis using SPSS and Stata 16 (StataCorp. 2019. Stata Statistical Software: Release 16. College Station, TX: StataCorp LLC). A P < 0.05 was considered statistical significance.

## Results

**Fig 1:**
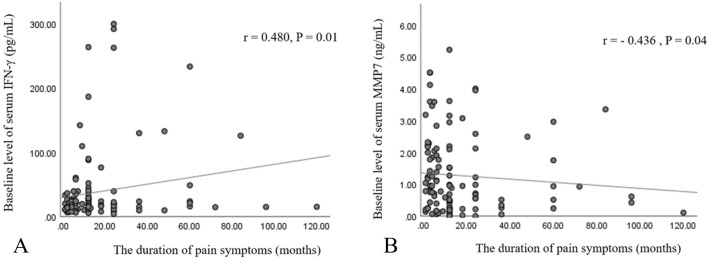
The correlation between the duration of pain symptoms and baseline serum inflammatory cytokine levels before TFESI. (A) IFN- γ, and (B) MMP-7. The lines of best fit are indicated on the graphs.

**Fig 2:**
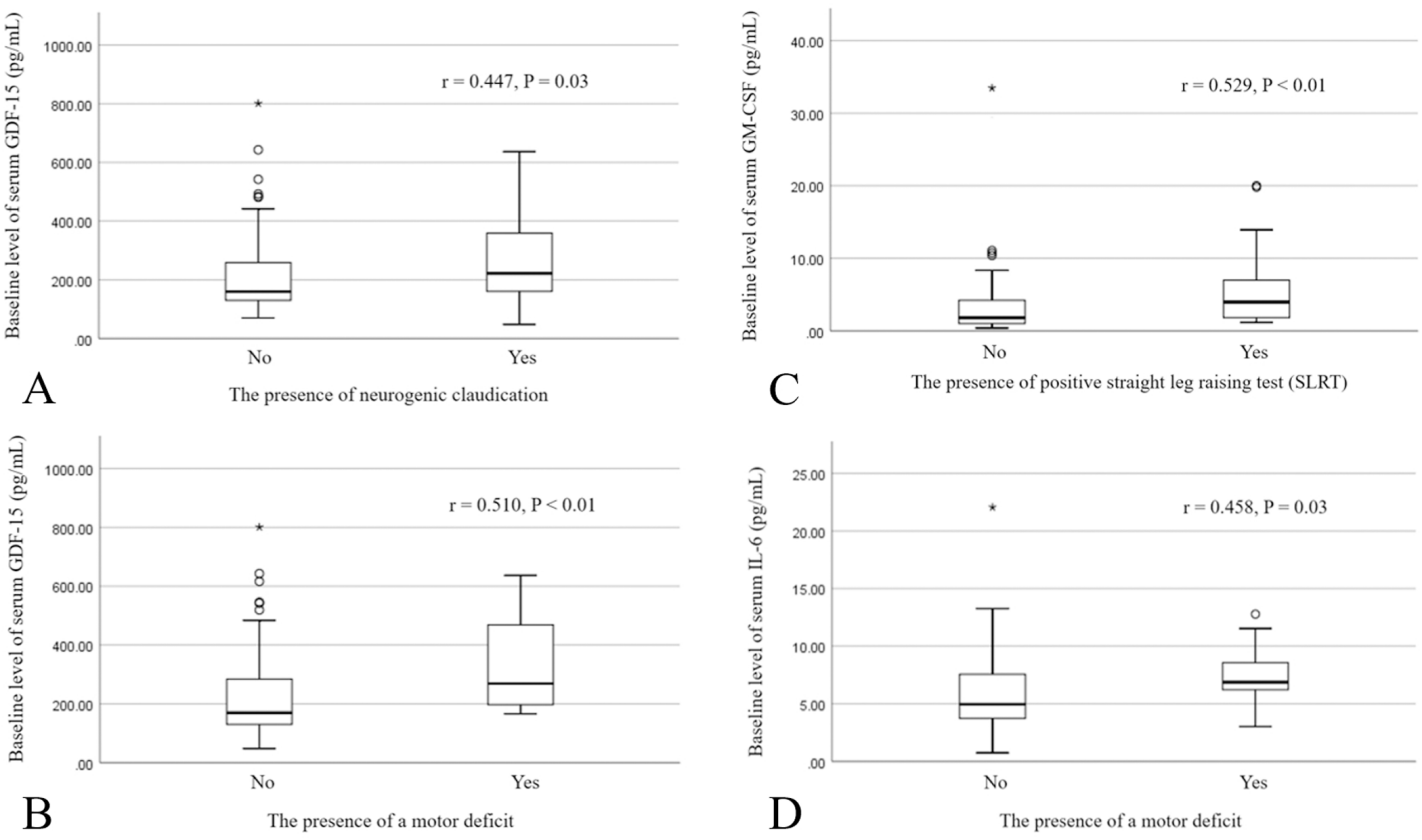
The correlation between clinical presentations and baseline serum inflammatory cytokines before TFESI. (A, B) GDF-15, (C) GM-CSF, and (D) IL-6.

The study enrolled 168 participants, excluding 48 individuals based on the exclusion criteria. Among those excluded, 23 had a history of lumbar surgery, 12 had recently undergone ESI, 5 exhibited symptoms of depression, 5 had systemic inflammatory disease, 2 were taking oral prednisolone, and 1 was diagnosed with a tumor involving epidural invasion. Clinical pain manifestation, patient characteristics, serum inflammatory cytokines, and MRI results were gathered from a cohort of 120 patients. Following two weeks after receiving TFESI, 73 out of 120 patients (60.8%) displayed a positive response, whereas 47 out of 120 patients (39.2%) exhibited unfavorable outcomes (Table 1). The levels of baseline serum inflammatory cytokines were comparable in both groups. Patients who showed positive responses exhibited a noteworthy decrease in serum levels of IFN-γ, IL-1β, IL-6, and GM-CSF two weeks after TFESI compared to their initial levels (P < 0.05) (Table 2). However, no statistically significant differences were observed in the mean changes of serum inflammatory cytokines between the two groups after TFESI (Table 2). There was a correlation between a longer duration of pain symptoms and elevated levels of IFN-γ (r = 0.480, P = 0.014), along with a reduction in MMP-7 levels (r = 0.436, P = 0.038) (Fig 1A, 1B). No significant correlation was found between the baseline serum levels of nine inflammatory cytokines and preoperative pain intensity or the presence of nerve root compression. Fig 2 depicts clinical pain manifestations that demonstrated a significant correlation with the levels of serum cytokines before TFESI. Neurogenic claudication, SLRT, and motor deficit exhibited a significant correlation with the increase in serum GDF-15, GM-CSF, and IL-6 and GDF-15, respectively (P < 0.05).

**Table 1:** Demographic data and baseline characteristics between good and poor responders to TFESI.

Variables	Good responder (n = 73)	Poor responder (n = 47)	P-value
**Age**	57.9 ± 15.3	57.0 ± 14.7	0.760
**Gender**: male/female	19 (26%)/54 (74%)	16 (34%)/31 (66%)	0.346
**BMI**	25.4 ± 5.5	27.9 ± 2.0	0.329
**Comorbidities**			
Diabetes Mellitus	12 (16.4%)	6 (12.8%)	0.293
Hypertension	39 (53.4%)	22 (46.8%)	0.242
Coronary artery disease	3 (4.1%)	4 (8.5%)	0.160
Chronic kidney disease	5 (6.8%)	5 (10.5%)	0.234
Osteoporosis	2 (2.7%)	0 (0.0)	0.128
**Primary diagnosis**			0.721
HNP	45 (61.6%)	27 (57.5%)	
Spinal canal stenosis	28 (38.4%)	20 (42.5%)	
**Level of TFESI**			0.790
L2-3	2 (2.7%)	2 (4.3%)	
L3-4	6 (8.2%)	3 (6.4%)	
L4-5	48 (65.8%)	34 (72.3%)	
L5-S1	17 (23.3%)	8 (17%)	
**Pain medications before TFESI**			
Gabapentinoids	68 (93.2%)	45 (95.7%)	0.279
SNRIs	6 (8.2%)	13 (27.7%)	0.002*
TCAs	5 (6.8%)	5 (10.6%)	0.234
Muscle relaxant	18 (24.7%)	14 (29.8%)	0.270
Weak opioids (tramadol/codeine)	56 (76.7%)	42 (89.4%)	0.041*
Strong opioids	1 (1.4%)	3 (6.4%)	0.069
**Pain characteristics**			
**Baseline pain intensity**			
Pain at rest (NRS 0–10)	5.9 ± 1.6	6.3 ± 2.0	0.250
Pain on movement (NRS 0–10)	8.1 ± 1.6	8.2 ± 1.6	0.968
**Duration of pain (months)**	13.9 ± 17.3	21.0 ± 25.9	0.102
**Baseline ODI**	42.1 ± 15.4	42.6 ± 16.6	0.858
**Clinical presentation**			
**Neurogenic claudication**	33 (45.2%)	18 (38.3%)	0.455
Maximum walking distance (meters)	181.7 ± 233.4	451.1 ± 162.1	0.344
**Positive SLRT**	18 (24.7%)	15 (31.9%)	0.385
**Presence of motor deficit**	6 (8.2%)	8 (17%)	0.143
**MRI findings**			
**The classification of disc herniation**			0.416
Bulging	47 (64.4%)	32 (68.1%)	
Protrusion	22 (30.1%)	10 (21.3%)	
Extrusion	4 (5.5%)	4 (8.5%)	
Sequestration	0 (0%)	1 (2.1%)	
**The location of disc herniation**			0.514
Central canal zone	33 (45.2%)	24 (51.1%)	
Subarticular zone	19 (26%)	8 (17%)	
Foraminal zone	21 (28.8%)	15 (31.9%)	
**The degree of central canal stenosis**			0.008*
Mild	42 (57.5%)	15 (31.9%)	
Moderate	26 (35.6%)	22 (46.8%)	
Severe	5 (6.8%)	10 (21.3%)	
**The degree of nerve root compromise**			0.064
Grade 1 (contact; normal position)	0 (0%)	0 (0%)	
Grade 2 (dorsal deviation)	29 (39.7%)	11 (23.4%)	
Grade 3 (compression)	44 (60.3%)	36 (76.6%)	
**The degree of lumbar foraminal stenosis**			0.537
Grade 1 (mild)	6 (8.2%)	6 (12.8%)	
Grade 2 (moderate)	59 (80.8%)	38 (80.8%)	
Grade 3 (severe)	8 (11.0%)	3 (6.4%)	
**Co-existing spondylolisthesis**	26 (35.6%)	19 (40.4%)	0.595

The value is presented as mean ± SD. and n (%).

HNP, herniated nucleus pulposus; TFESI, transforaminal epidural steroid injection; SNRIs, serotonin noradrenaline reuptake inhibitors; TCAs, tricyclic antidepressants; NRS, numeric rating scale; ODI, Oswestry Disability Index; SLRT, straight leg raising test; MRI, magnetic resonance imaging.

*Statistically significant difference.

**Table 2:** The serum concentrations of cytokines between good responders and poor responders to TFESI.

Cytokines		Good responder (n = 73)	Poor responder (n = 47)	P-value
CRP (ng/mL)	Pre-TFESI	1845.8 ± 1478.7	1719.0 ± 1419.9	0.642
	Post-TFESI	1664.5 ± 1417.2	1574.0 ± 1299.6	0.725
	Mean change	181.3 ± 1462.1	145.0 ± 1004.0	0.882
	*p*-value (within a group)	0.293	0.327	
IFN-γ (pg/mL)	Pre-TFESI	36.5 ± 54.9	40.9 ± 62.9	0.689
	Post-TFESI	29.5 ± 41.4	38.3 ± 47.9	0.295
	Mean change	7.0 ± 25.4	2.6 ± 26.9	0.487
	*p*-value (within a group)	0.025*	0.504	
IL-1β (pg/mL)	Pre-TFESI	4.4 ± 4.9	4.8 ± 5.6	0.695
	Post-TFESI	3.6 ± 4.1	4.6 ± 5.7	0.293
	Mean change	0.8 ± 2.3	0.1 ± 3.4	0.457
	*p*-value (within a group)	0.029*	0.723	
IL-6 (pg/mL)	Pre-TFESI	6.0 ± 3.3	5.8 ± 2.8	0.667
	Post-TFESI	5.0 ± 2.3	5.1 ± 3.0	0.818
	Mean change	1.1 ± 2.9	0.7 ± 2.8	0.880
	*p*-value (within a group)	0.003*	0.117	
TNF-α (pg/mL)	Pre-TFESI	5.4 ± 7.6	4.1 ± 6.5	0.349
	Post-TFESI	4.5 ± 4.9	4.9 ± 5.9	0.682
	Mean change	0.9 ± 9.0	0.82 ± 9.4	0.603
	*p*-value (within a group)	0.416	0.561	
GM-CSF (pg/mL)	Pre-TFESI	7.2 ± 15.3	5.6 ± 11.2	0.528
	Post-TFESI	1.7 ± 2.6	4.7 ± 21.4	0.331
	Mean change	5.6 ± 15.4	0.9 ± 24.7	0.200
	*p*-value (within a group)	0.003*	0.812	
MMP-7 (ng/mL)	Pre-TFESI	1.3 ± 1.2	1.3 ± 1.2	0.899
	Post-TFESI	1.5 ± 1.1	1.4 ± 1.3	0.768
	Mean change	0.2 ± 1.3	0.2 ± 1.2	0.806
	*p*-value (within a group)	0.216	0.396	
VEGF (pg/mL)	Pre-TFESI	125.5 ± 84.4	154.3 ± 127.7	0.149
	Post-TFESI	131.5 ± 82.2	143.5 ± 82.1	0.447
	Mean change	6.0 ± 72.4	10.8 ± 131.2	0.180
	*p*-value (within a group)	0.490	0.585	
GDF-15 (pg/mL)	Pre-TFESI	227.7 ± 138.6	250.6 ± 164.5	0.417
	Post-TFESI	212.1 ± 114.2	234.5 ± 146.6	0.385
	Mean change	15.5 ± 94.2	16.1 ± 86.8	0.362
	*p*-value (within a group)	0.163	0.219	

The value is presented as mean ± SD. and n (%).

CRP, C-reactive protein; IFN- γ, interferon-gamma; IL-1β, interleukin-1beta; IL-6, interleukin-6; TNF-α, tumor necrosis factor-alpha; GM-CSF, granulocyte-macrophage colony-stimulating factor; MMP-7, matrix metalloproteinase 7; VEGF, vascular endothelial growth factor; GDF-15, growth differentiation factor 15; ng/mL, nanograms per milliliter; pg/mL, picograms per milliliter; TFESI, transforaminal epidural steroid injection.

*Statistically significant difference.

The MRI findings in both groups were comparable, except for differences observed in the degree of central canal stenosis (Table 1). Individuals who exhibited a poor response to TFESI were found to have severe central canal stenosis, whereas those who responded favorably demonstrated a significant predominance of mild central canal stenosis (P = 0.008). The elevated level of serum IFN-γ demonstrated a significant correlation with a greater degree of HNP (r = 0.436, P = 0.039) while the increased serum concentration of MMP-7 exhibited a significant correlation with the presence of foraminal stenosis (r = 0.436, P = 0.045). The presence of nerve root compression was correlated with higher pain intensity post-ESI at 2 weeks (r = 0.478, P = 0.012) and 1 month (r = 0.440, P = 0.034) as well as higher disability score at 2 weeks (r = 0.526, P = 0.002) and 1 month (r = 0.491, P = 0.008). The results of the univariate and multivariate logistic regression analyses are presented in Table 3. The univariate analysis identified several factors associated with a positive response, including the degree of central canal stenosis and nerve root compromise on MRI. However, the multivariate analysis revealed that only a mild degree of central canal stenosis significantly predicted a favorable response to TFESI.

**Table 3:** Univariate and multivariate analysis of variables associated with good and poor responders to TFESI.

	Univariate		Multivariate	
	OR (95%CI)	P-value	Adjusted OR (95%CI)	P-value
**Age**	1.004 (0.98, 1.029)	0.758		
**Gender**				
1	Reference	1		
2	1.467 (0.66, 3.259)	0.347		
**BMI**	0.985 (0.952, 1.019)	0.389		
**Level of TFESI**				
L2-3	0.471 (0.056, 3.97)	0.488		
L3-4	0.941 (0.186, 4.759)	0.942		
L4-5	0.664 (0.257, 1.715)	0.398		
L5-S1	Reference	1		
**Clinical presentations**				
**Duration of pain**	0.984 (0.967, 1.002)	0.086	0.985 (0.967, 1.004)	0.122
**Neurogenic claudication**				
Yes	Reference	1		
No	0.752 (0.356, 1.588)	0.455		
**Maximum walking distance**	0.999 (0.998, 1.001)	0.323		
**Positive SLRT**				
Yes	Reference	1		
No	1.432 (0.636, 3.226)	0.386		
**Presence of motor deficit**				
Yes	Reference	1		
No	2.291 (0.74, 7.089)	0.150		
**MRI findings**				
**The classification of disc herniation**				
Bulging	Reference	1		
Protrusion	1.498 (0.626, 3.583)	0.364		
Extrusion	0.681 (0.159, 2.923)	0.605		
Sequestration	0 (0, 1)	1		
**The location of disc herniation**				
Central canal zone	Reference	1		
Subarticular zone	1.727 (0.649, 4.599)	0.274		
Foraminal zone	1.018 (0.437, 2.372)	0.967		
**The degree of central canal stenosis**				
Mild	5.6 (1.645, 19.058)	0.006*	4.827 (1.238, 18.817)	0.023*
Moderate	2.364 (0.702, 7.961)	0.165	2.225 (0.648, 7.636)	0.204
Severe	Reference	1	Reference	1
**The degree of nerve root compromise**				
Grade 1 (normal position)	NA	1		
Grade 2 (dorsal deviation)	2.157 (0.948, 4.907)	0.067	1.195 (0.444, 3.216)	0.725
Grade 3 (compression)	Reference	1	Reference	1
**The degree of lumbar foraminal stenosis**				
Grade 1 (mild)	0.375 (0.066, 2.145)	0.270		
Grade 2 (moderate)	0.582 (0.145, 2.333)	0.445		
Grade 3 (severe)	Reference	1		
**Co-existing spondylolisthesis**				
Yes	Reference	1		
No	1.23 (0.58, 2.61)	0.596		
**Cytokine levels**				
**CRP** pre-TFESI	1 (0, 1)	0.838		
post-TFESI	1 (0, 1)	0.858		
mean difference	1 (0, 1)	0.960		
**IFN-γ** pre-TFESI	0.999 (0.992, 1.005)	0.687		
post-TFESI	0.996 (0.987, 1.004)	0.299		
mean difference	0.993 (0.977, 1.009)	0.381		
**IL-1β** pre-TFESI	1.015 (0.943, 1.092)	0.693		
post-TFESI	1.045 (0.962, 1.137)	0.297		
mean difference	1.076 (0.944, 1.227)	0.274		
**IL-6** pre-TFESI	1.028 (0.907, 1.166)	0.664		
post-TFESI	0.983 (0.848, 1.139)	0.816		
mean difference	0.953 (0.829, 1.095)	0.496		
**TNF-α** pre-TFESI	1.028 (0.969, 1.091)	0.353		
post-TFESI	0.985 (0.918, 1.057)	0.680		
mean difference	0.979 (0.938, 1.022)	0.332		
**GM-CSF** pre-TFESI	1.024 (0.959, 1.093)	0.480		
post-TFESI	1.004 (0.851, 1.184)	0.965		
mean difference	0.979 (0.921, 1.042)	0.506		
**MMP-7** pre-TFESI	1.021 (0.748, 1.393)	0.898		
post-TFESI	1.049 (0.766, 1.435)	0.765		
mean difference	1.026 (0.757, 1.391)	0.869		
**VEGF** pre-TFESI	0.997 (0.994, 1.001)	0.156		
post-TFESI	0.998 (0.994, 1.003)	0.444		
mean difference	1.002 (0.998, 1.006)	0.382		
**GDF-15** pre-TFESI	0.999 (0.996, 1.001)	0.415		
post-TFESI	0.999 (0.996, 1.002)	0.354		
mean difference	1 (0.996, 1.004)	0.973		

HNP, herniated nucleus pulposus; TFESI, transforaminal epidural steroid injection; SLRT, straight leg raising test; MRI, magnetic resonance imaging; CRP, C-reactive protein; IFN- γ, interferon-gamma; IL-1β, interleukin-1beta; IL-6, interleukin-6; TNF-α, tumor necrosis factor-alpha; GM-CSF, granulocyte-macrophage colony-stimulating factor; MMP-7, matrix metalloproteinase 7; VEGF, vascular endothelial growth factor; GDF-15, growth differentiation factor 15.

*Statistically significant difference.

All patients were followed up for 12 months. Patients classified as good responders encountered a significant 29-week period of reduced pain (95% confidence interval 10.3–47.8) following a single TFESI. Moreover, they showed a significantly decreased probability of requiring surgery at the 12-month follow-up, with only 10 out of 73 patients (13.7%) undergoing spinal surgery (P < 0.001). Patients categorized as poor responders reported minimal pain relief, typically lasting no longer than one week. After one year, a substantial proportion of these patients (24 out of 47; 51.1%) ultimately needed surgery.**Discussion**

This prospective analytical study was the first to investigate the association of clinical manifestations, MRI results, and serum inflammatory cytokines in patients with lumbosacral radiculopathy. Patients who exhibited a favorable response to TFESI showed a remarkable decrease in serum levels of IFN-γ, IL-1β, IL-6, and GM-CSF at two weeks compared to their initial baseline measurements. The elevated level of serum IFN-γ showed a significant correlation with a greater degree of disc herniation, whereas the increased serum concentration of MMP-7 was significantly associated with the presence of foraminal stenosis.

Degenerative conditions of intervertebral discs and spinal canal stenosis typically exhibit amplified matrix degradation, angiogenesis, nerve innervation, and heightened expression of catabolic cytokines [7]. As a result, many studies demonstrated that increases in serum inflammatory cytokines, including IL-1β, IL-6, and TNF-α, were found in acute and chronic low back pain patients [15–17]. However, there is limited data on the use of these cytokines for predicting treatment responses. The current study found that patients who displayed a positive response to TFESI showed a significant decrease in serum IFN-γ levels when compared to their baseline measurement. The results aligned with the findings of a previous study by Scuderi GJ, which revealed that a substantial reduction of IFN-γ in the epidural lavage fluid indicated pain reduction three months after a caudal ESI [8]. Moreover, this study also found that the higher level of serum IFN-γ before TFESI demonstrated a significant correlation with the increased severity of HNP and the longer duration of pain symptoms. Prior research documented the infiltration of macrophages and lymphocytes, along with the expression of IFN-γ and IL-6, in herniated intervertebral disc tissues [18]. The results from this study and previous research highlighted the potential role of IFN-γ as a predictive biomarker for a favorable response to ESI in patients with lumbosacral radiculopathy. IFN-γ serves as a pivotal inflammatory cytokine, primarily activating macrophages and monocytes, subsequently leading to the release of various inflammatory mediators. It may be involved in sustaining these immune responses, which can contribute to persistent pain. The present study also illustrated reduced serum levels of IL-1β, IL-6, and GM-CSF in patients who reported a positive response to TFESI. These findings were consistent with a prior study in chronic back pain patients showing that the levels of inflammatory mediators were lower following the medical treatments [19]. In clinical practice, serum hs-CRP is frequently employed as an indicator of inflammation and tissue injury. However, the serum level of hsCRP remained unchanged in patients with chronic low back pain or radiculopathy [20]. The research conducted by Park et al. revealed no significant correlation between serum hs-CRP levels and pain scores [21]. Furthermore, hs-CRP was not a reliable marker for predicting the efficacy of TFESI [22]. Consistent with this study, hs-CRP exhibited no association with pain scores and did not serve as a biomarker for identifying favorable responders to TFESI.

This study furnishes additional insights into the heightened levels of specific inflammatory cytokines and their correlation with distinct clinical manifestations and the severity of MRI findings in individuals afflicted with lumbosacral radiculopathy. The results demonstrated that the higher level of serum IFN-γ significantly correlated with a greater degree of HNP, while the elevated serum concentration of MMP-7 exhibited a significant correlation with the presence of foraminal stenosis. MMP-7 plays a role in osteoclastic activity and bone remodeling [23]. Its increased activity may contribute to osteophyte formation (bone spurs) around the foramen, leading to inflammation and nerve compression. This can result in fibrosis and hypertrophy of the ligamentum flavum, ultimately causing foraminal stenosis. Moreover, the duration of pain symptoms was significantly correlated with the decreased level of MMP-7. Matrix metalloproteinases (MMP) likely play a pivotal role in the degradation of intervertebral discs and subsequently disc resorption [24,25]. Abundant expression of MMP-7 was found in mononuclear cells, chondrocytes, and intervertebral disc tissue among patients undergoing surgery, while normal-appearing discs exhibited minimal expression [26]. Moreover, the expression of MMP-7 significantly increased with the greater degree of disc herniation into the epidural space [26]. In an animal study, following the administration of recombinant human matrix metalloproteinases-7 (rhMMP-7) into the herniated disc or the epidural space, there was a significant reduction in disc protrusion observed through MRI and myelography after 7 days [27,28], suggesting its potential as a promising chemonucleolysis agent for treating herniated discs. Thus, these emphasize the essential role of macrophage-derived MMP-7 in disc resorption and the invasion of disc tissue by macrophages. This study was the first to establish the role of MMP-7 in spinal pathology and low back pain in humans. This study also found no correlation between pain severity and serum cytokine levels, which is consistent with a recent systematic review [15]. These findings suggest that pain may not solely be attributable to heightened inflammatory processes but rather could also be influenced by other factors, such as pain experience and psychosocial aspects.

Beyond changes in biomarkers, this study also demonstrated clinically meaningful improvements in objective functional outcomes. Patients who responded well to TFESI not only exhibited reductions in pro-inflammatory cytokines but also experienced greater enhancements in disability scores (ODI), longer durations of pain relief, and significantly lower surgery rates at 12 months (13.7% vs. 51.1%, P < 0.001). Notably, the improvements in ODI scores aligned with reductions in pain intensity, further supporting the connection between functional recovery and symptomatic relief following TFESI. These findings suggest a potential link between early immunological shifts and long-term functional benefits, though further research is necessary to validate these associations.

The strength of this study was its ability to establish connections between different cytokines and clinical pain manifestations, pain severity, and MRI findings. The magnitude of biomarker change post-TFESI may serve as an indicator of long-term improvement. Patients with significant reductions in serum IFN-γ, IL-1β, IL-6, and GM-CSF levels after TFESI exhibited more sustained responses, while minimal changes may indicate a transient or short-term outcome. This differentiation can help identify individuals who might benefit from additional injections or alternative treatments, such as surgery. However, established serum cytokine thresholds are necessary to guide clinical decision-making.

The limitations of this study were the absence of laboratory results after TFESI beyond 2 weeks and the comparable data between the serum and epidural cytokine levels. Hence, the outcomes would reflect the applicability for a short-term duration among good responders. Furthermore, there was no evaluation of serum cytokine levels between patients with lumbosacral radiculopathy and healthy participants serving as a control group. Our primary focus was on predicting a positive response to TFESI rather than defining diagnostic thresholds or disease-specific profiles. Moreover, TFESI cannot be administered to healthy participants. Another limitation was the use of standard inflammatory cytokines for analysis. There could be other cytokines potentially involved in the spinal disorders that were not assessed. Cytokine levels exhibit diurnal variations, and since blood samples were not collected at specific time points, this was impractical. Additionally, the usefulness of the serum cytokines as predictive factors after TFESI was questionable in clinical settings, although it provided further evidence regarding the role of inflammatory components in degenerative spinal pain. Correlations with R values below 0.5 are considered weak and require further investigation. While certain biomarkers demonstrated statistically significant changes, the modest effect sizes observed may limit their biological or clinical relevance, and further validation is warranted. Another limitation was the small sample size, which required more patients with a favorable response to enable a robust logistic regression analysis. Lastly, serum cytokine levels may not precisely represent epidural cytokine concentrations, which could better reflect local inflammation. However, obtaining epidural fluid through epidural lavage presents technical challenges. Epidural lavage using a larger-bore needle, such as a Tuohy needle, via the caudal space is sufficient to access lower lumbar (L4-5) pathologies, however, it may not adequately reach upper lumbar (L1-3) pathologies.

## Conclusions

The decrease in serum IFN-γ, IL-1β, IL-6, and GM-CSF levels post-TFESI was a promising biomarker for a favorable response, highlighting the role of inflammatory components in lumbosacral radiculopathy. Cytokines with promising potential for significant involvement in clinical presentations included IFN-γ, MMP7, IL-6, GDF-15, and GM-CSF. Further research is warranted to elucidate these findings. Clinical manifestations, diagnostic imaging, and laboratory results must be carefully evaluated to determine a favorable response to TFESI.

## Supporting information

S1 Supplementary results_detailed. (XLSX)

S2 Minimal data set. (XLSX)
